# Quantitative sensory testing and classical pain model dataset in 127 healthy volunteers

**DOI:** 10.1016/j.dib.2026.112842

**Published:** 2026-05-12

**Authors:** Jörn Lötsch, Violeta Dimova, Isabel Lieb, Dario Kringel

**Affiliations:** a Goethe University, Institute of Clinical Pharmacology, Faculty of Medicine, Theodor-Stern-Kai 7, 60590 Frankfurt am Main, Germany; b University of Helsinki, Faculty of Medicine, Haartmaninkatu 8, P.O. Box 63, Helsinki 00014 Finland; c Fraunhofer Institute for Translational Medicine and Pharmacology (ITMP), Theodor-Stern-Kai 7, 60596 Frankfurt am Main, Germany

**Keywords:** Psychophysics, Human, Pain research, Experimental pain

## Abstract

Experimental pain models are integral to human pain research and the development of analgesic drugs. Of many options available, it is possible to identify pain models that reliably predict clinical analgesic efficacy in cost-effective laboratory settings. However, translating experimental findings to clinical practice is imperfect, and laboratory models can only partially replicate specific clinical pain conditions. The present dataset originates from a study that addressed this gap between experimental and clinical pain. The broader study investigated whether patterns of clinical neuropathic pain could be induced, at least to some degree, in healthy human subjects using topical capsaicin application to induce hypersensitization. This dataset provides the baseline control measurements from untreated skin that were obtained as part of this research.

The study enrolled 127 healthy volunteers who underwent comprehensive sensory testing. A quantitative sensory testing (QST) battery developed for clinical diagnosis by the German Research Network on Neuropathic Pain was utilized to characterize baseline sensory function and pain sensitivity.

The dataset is provided in both its original (raw) and transformed formats. Key pain data are presented as a 127×23 matrix (127 cases and 23 QST variables). Transformations include sign inversion for certain variables to align pain sensitivity, directionality, unit conversions, and log transformations based on psychophysical principles. A separate metadata file documents participant characteristics, such as age, sex, test site allocation (hand or foot), and body side tested.

This dataset has significant potential for reuse. Researchers can use it to benchmark new pain assessment tools, validate experimental pain models, and develop or test statistical and computational methods for analyzing sensory and pain data. The inclusion of demographic variables allows for the investigation of how age and sex influence pain perception. The structure of the dataset supports secondary analyses, integration into machine learning workflows as independent validation data, and use as a reference in future pain research. All data are anonymized and formatted to facilitate reproducibility and secondary use.

Specifications Table

**Table:** 

Subject	Health Sciences, Medical Sciences & Pharmacology
Specific subject area	Specific subject area: Quantitative sensory testing and experimental pain model data in healthy adults from untreated (control) skin sites.
Type of data	Table, Raw, Processed, Comma-separated values (csv)
Data collection	Healthy adults underwent standardized quantitative sensory testing (QST) and classical pain models. The instruments used were the Medoc TSA-II (thermal), the JTECH Commander Algometer (pressure), the Neurometer CPT (electrical), the Burghart OM/2 Olfactometer (CO₂), and the StarMedTec Themis Laser (laser pain). Inclusion criteria: no chronic pain. The data were normalized via log transformation, unit conversion, and sign inversion for pain sensitivity.
Data source location	Goethe - University, Frankfurt am Main, Germany
Data accessibility	Repository name: Mendeley Data Data identification number: doi: 10.17632/9v8ndhctvz.2 Direct URL to data: https://data.mendeley.com/datasets/9v8ndhctvz/2 Instructions for accessing these data: Dataset name in Mendeley data: “Quantitative sensory testing and classical pain model dataset in 127 healthy volunteers”. Data can be downloaded as three comma-separated values (CSV) files.
Related research article	Lötsch J, Dimova V, Ultsch A, Lieb I, Zimmermann M, Geisslinger G, Oertel BG. A small yet comprehensive subset of human experimental pain models emerging from correlation analysis with a clinical quantitative sensory testing protocol in healthy subjects. Eur J Pain. 2016 May;20(5):777–89. doi: 10.1002/ejp.803.

## 1 Value of the Data

• Comprehensive multimodal pain data: This dataset contains raw and processed data from various pain assessment methods in humans. It includes components of a standardized clinical quantitative sensory testing (QST) battery, as well as pain-related measures acquired using “classical” experimental human pain models.
• Various pain-related sensations: The included measures comprise sensory functions relevant to pain, such as pain thresholds, pain tolerance, and subjective pain intensity. Nociceptive stimuli include various physical modalities, such as mechanical, thermal, electrical, and chemical stimuli. These features qualify the data set for research on human pain physiology.
• Standardized and reproducible protocols: All data were collected using standardized methods, including the QST battery from the German Research Network on Neuropathic Pain, as well as validated and well-described laboratory protocols on classical human experimental pain models. These protocols ensure reproducibility and comparability with other studies. These features qualify the dataset for meta-analyses and the development of normative reference values.
• Canonical data for method development in pain research: The dataset can also be used for benchmarking new pain assessment tools and validating experimental pain models.
• Comprehensive pain data for machine learning: This dataset is suitable for developing or testing statistical and computational methods in human sensory pain research. Its structure and level of detail allow for secondary analyses, integration into machine learning workflows, and exploration of new data analysis approaches. The dataset may qualify as independent validation data for machine-learning based analysis of quantitative sensory studies.
• Support for research on demographic factors in pain perception: This dataset is ideal for studying demographic factors in pain perception, particularly sex differences and age-related changes, both areas of growing interest in clinical and basic pain research. The cohort comprises younger Caucasian adults (age range 18–36 years, mean ± SD: 25 ± 3.1 years), making it particularly suitable for comparison studies in demographically matched cohorts and for investigating pain perception within this specific age range. The well-defined demographic characteristics enable researchers to control for age and ethnicity variables when using these data as reference or validation datasets.

## 2 Background

This dataset originates from a study in which quantitative sensory testing (QST) was performed on healthy volunteers to characterize baseline sensory function related to pain perception and, in a separate experimental condition, evaluate capsaicin-induced sensitization [1]. This dataset contains control measurements from untreated skin. However, the data were acquired as part of a project that also assessed sensitization following topical capsaicin application. The results from the first 100 subjects were published separately [2]. The broader study goal was to develop predictive experimental human pain models to support the development and optimization of pain therapies.

This set of models is widely used in clinical and pharmacological research on pain and analgesia to investigate the effects of analgesic drugs under standardized conditions. While such models allow for rather valid predictions of clinical effects of analgesics [3,4], certain conditions such as neuropathic pain still pose challenges for the prediction of clinical drug effects. To better mirror clinical pain states, a standardized QST battery [5,6] was used alongside classical pain models to record comprehensive sensory profiles. The present dataset provides the baseline sensory profiles from untreated skin.

QST values from untreated skin generally matched reference ranges, providing a normative baseline for comparison. In the broader study, capsaicin-sensitized measurements (published separately) showed deviations in several parameters; some subjects showed substantial resemblance to neuropathic profiles, with significant sex differences [7].

## 3 Data Description

The dataset includes three comma-separated values (CSV) files, each of which plays a specific role in representing the pain and sensory testing data and associated metadata.

### 3.1 qst_pain_data_orig.csv

This file contains the original QST data matrix, which has 127 rows and 23 columns. The first column (“ID”) lists arbitrary subject identifiers. Columns two through 23 contain various measures related to pain sensitivity. These include pain thresholds, which are the smallest physical strength of a nociceptive stimulus that evokes the sensation of pain, or pain tolerance values, which represent the highest intensity of the nociceptive stimuli that could be tolerated by the study participants during the experiments. Occasionally, this reached the technical limit of the stimulation device. They also include pain intensity estimates on visual analog scales.

The first set of QST parameters were acquired using well-established and frequently used experimental human pain models. These models are summarized here under the heading “classical” pain models. The details are provided in Table 1.

**Table 1:** So-called “classical” pain models are a collection of additional QST measures that have been established in experimental human pain research. However, they are not redundant or performed differently than the above-standardized clinical QST test battery. The values provided in the files were obtained after the basic data processing described in the fourth column of the table.

Test	Variable	Sensory dimension	Basic data processing	Unit
Blunt mechanical pressure	**PressureThr**	Mechanical pain threshold (blunt pressure)	Median of 5 threshold measurements (pressure at first pain) at dorsal side of mid-phalanx of right middle finger	N/cm²
	**PressureTol**	Mechanical pain tolerance (blunt pressure)	Median of 5 tolerance measurements (pressure at intolerable pain) at dorsal side of mid-phalanx of right middle finger	N/cm²
Thermal pain	**TSACold**	Cold pain threshold (thermal)	Median of 5 threshold measurements (temperature at first pain) on medial volar forearm	°C
	**LaserThr**	Heat pain threshold (infrared laser)	Mean of highest non-painful and lowest painful pulse energy (10 stimuli, staircase method) on dorsum of left hand	mJ
	**LaserVAS**	Heat pain intensity (infrared laser)	Mean VAS rating of 23 stimuli at 650 mJ (0 - 100 rating scale) on dorsum of left hand	mm
Electrical stimulation	**ElectricThr**	Electrical pain threshold	Median of 5 threshold measurements (current at first pain). The electrodes were placed at the medial and lateral side of the left mid-phalanx (middle finger of the left hand as default-testing site)	mA
	**ElectricTol**	Electrical pain tolerance	Median of 5 tolerance measurements (current at intolerable pain) at left mid-phalanx	mA
Chemical (intranasal CO_2_)	**CO2Thr**	Chemical pain threshold (CO_2_)	Mean of highest non-painful and lowest painful CO_2_ concentrations (16 stimuli, randomized order)	% v/v
	**CO2VAS**	Chemical pain intensity (CO_2_)	Mean VAS rating of 23 stimuli at 65% v/v CO_2_ (0 - 100 rating scale)	mm

*Note*: In the transformed data file (qst_pain_data_transformed.csv), pressure values (PressureThr and PressureTol) are converted from N/cm² to kPa (kilopascal).

Furthermore, thirteen sensory measures from the Quantitative Sensory Testing (QST) battery were included. This battery was developed by the German Research Network on Neuropathic Pain for clinical diagnosis of neuropathic pain [5,6]. Details about these parameters are provided in Table 2.

**Table 2:** Components of the quantitative sensory testing battery of the German Research Network on Neuropathic Pain. Table adapted from [2]. The values provided in the files were obtained after the basic data processing described in the fourth column of the table.

Test	Variable		Sensory dimension	Basic data processing	Unit
Thermal testing	**CDT**	Cold detection threshold	Topical application of cold stimuli, baseline t° = 32 °C, decreasing by 1 °C/s	Difference from baseline 32 °C of the mean of 3 measurement repetitions, log*-transformation*	°C-32
	**WDT**	Warm detection threshold	Topical application of warm stimuli, baseline t° = 32 °C, increasing by +1 °C/s	Difference from baseline 32 °C of the mean of 3 measurement repetitions, log*-transformation*	°C-32
	**TSL**	Thermal sensory limen	Topical application of alternating cold and warm stimuli	Difference in the means of the 3 warmth and the 3 cold detection thresholds, log-transformation	Δ °C
	**PHS**	Paradoxical heat sensations	Number of paradoxical heat sensations recorded during TSL	Number of paradoxical heat sensations, max. 3	count
	**CPT**	Cold pain threshold	Topical application of cold stimuli, baseline t° = 32 °C; decreasing by 1 °C/s	Mean of the 3 measurement repetitions	°C
	**HPT**	Heat pain threshold	Topical application of warm stimuli, baseline t° = 32 °C, increasing by 1 °C/s	Mean of the 3 measurement repetitions	°C
Pressure pain threshold	**PPT**	Pressure pain threshold	Application of blunt pressure stimuli to *M. thenar* for the hand area and *M. abductor hallucis* for the foot area	Mean of the 3 measurement repetitions, log-transformation	kPa
Mechanical pain threshold	**MPT**	Mechanical pain threshold	Application of pinprick stimuli (8 to 512 mN; contact area 0.2 mm) following staircase paradigm, starting force of 8 mN	Geometric mean of the 5 ascending and 5 descending stimuli, log-transformation	mN
Stimulus-response function	**MPS**	Mechanical pain sensitivity for pinprick stimuli	Application of pinprick stimuli and tactile stimuli in a balanced order, 0 - 100 pain rating scale.	Geometric mean of the pain ratings of the 35 pinprick stimuli, log-transformation	mm
	**DMA**	Dynamic mechanical allodynia for stroking light touch	Application of pinprick stimuli and tactile stimuli (cotton wist, cotton wool tip, standardized brush), 0 - 100 pain rating scale.	Geometric mean of the pain ratings of the 15 tactile stimuli, log-transformation	mm
Wind-up	**WUR**	Wind-up ratio	Temporal summation of pinprick stimuli, application of single pinprick stimulus (256 mN) followed by train of 10 pinprick stimuli (256 mN, 1/s repetition rate), 0 - 100 pain rating scale	Ratio of the mean pain ratings of the 5 series of stimuli and the mean pain ratings of the 5 single stimuli, log-transformation	unitless
Mechanical detection threshold	**MDT**	Mechanical detection threshold	Application of von Frey hairs (forces 0.25 to 512 mN, diameter 0.5 mm), staircase paradigm, starting force of 16 mN	Geometric mean of the 5 ascending and 5 descending stimuli, log-transformation	mN
Vibration detection threshold	**VDT**	Vibration detection threshold	Application of descending vibration stimuli (64 Hz, 8/8 scale) to the *Processus styloideus radii* for hand area and *Malleolus medialis* for foot area	Mean of the 3 measurement repetitions	scale units (0–8)

### 3.2 qst_pain_data_transformed.csv

This file contains the same 127 cases and 23 columns as the previous file, but it provides the QST values after a couple of basic transformations. One such transformation is sign-inversion (multiplication by −1), which ensures that higher values indicate greater pain sensitivity for all pain-related variables. This applies to the variables TSACold, CO2VAS, LaserVAS, CDT, CPT, MPS, WUR, VDT, and DMA. For example, for heat stimuli, higher pain sensitivity is indicated by a lower temperature at which subjects report pain. Conversely, for cold stimuli, higher pain sensitivity is indicated by a higher temperature. As the cold stimulus gradually decreased, the temperature at which the subject indicated pain increased with greater sensitivity. Therefore, the directions of certain OST variables were reversed. Second, for blunt pressure stimuli, the device-indicated unit of N/cm² was converted to the standard kPa (kilopascal). Third, the rotated and unit-transformed data were log transformed according to the law of Weber and Fechner for psychophysical measures [8]. Details of the data processing from file 1 to file 2 can be found at https://github.com/JornLotsch/experimental-pain-models-qst, with the corresponding archived release accessible via Zenodo at https://doi.org/10.5281/zenodo.18893795.

### 3.3 qst_pain_metadata.csv

This metadata file has 127 rows and five columns. The first column lists subject IDs, which are consistent with those in the other files. The second column provides the subjects’ age in years. Columns three through five provide three possible classification scenarios:

• “Sex,” coded as 1 for male and 2 for female.
• “QST_areal_1hand2foot”: The body area where the QST test was performed.
• “Test_side_1r2l”: The body side where the control measurements were performed. Note: The original study design included capsaicin testing on the contralateral side, which is not included in this dataset.

All files maintain consistent row order by subject ID, facilitating cross-referencing.

## 4 Experimental Design, Materials and Methods

The dataset was collected as part of a project to develop predictive human experimental pain models. The study included 127 healthy Caucasian volunteers (59 men) by self-assignment, aged between 18 and 36 years (mean ± SD: 25 ± 3.1 years). Participants were recruited via flyers placed at student meeting points throughout Campus Niederrad at Goethe University in Frankfurt am Main, Germany. Reimbursement of €150 was provided for the full study, which in addition to the present data also included a sensitization condition as mentioned above. Inclusion criteria had been age ≥ 18 years and no relevant current medical history. Health status was confirmed through medical history, physical examination, and assessment of vital signs. Exclusion criteria included drug intake during the previous week (except for oral contraceptives and vitamin or hormone substituting drugs), current clinical conditions involving pain, and current diseases according to questioning and medical examination. Participants were instructed to refrain from analgesic medications for at least one week prior to testing. Smoking, caffeine, and exercise were not recorded or controlled for.

Each participant attended a screening and training session (approximately 1 to 1.5 h), followed by a testing day approximately 20 h later (approximately 3 to 3.5 h). During the testing day, the two sessions were administered in a fixed order: the standardized QST battery was completed first, followed by the classical experimental pain test session [1]. A 30-minute rest period separated the two sessions, during which participants were seated quietly. The QST protocol was applied according to the original instructions [5,6], which state that the full battery is designed to provide a complete sensory profile for one body region within approximately 27 to 30 min per area. The classical pain test session was estimated to take 45 to 60 min [1], yielding interstimulus intervals of approximately 1 to 2 min between individual tests within that session. This is well above the range associated with habituation of chemosensory pain responses, which have been shown to decline markedly at ISIs of 10 to 20 s [9]. All measurements in this dataset were obtained from untreated skin (control sites). The broader study also included capsaicin-sensitized measurements on the contralateral limb, but these are not included in the present dataset and have been published separately. All measurements were performed by two trained investigators in accordance with the published instructions [5,6], which were also reported in [2] for the present assessments. Each participant was assessed by the same investigator across all sessions, ensuring intra-subject consistency; however, investigator identity was not recorded, precluding formal assessment of inter-investigator variability.

### 4.1 Classical experimental pain models

Classical experimental pain models were also used regardless of the QST battery. These tests involved applying mechanical, thermal, electrical, and chemical stimuli. All procedures were performed according to established laboratory protocols. See Table 1 for further details.

#### 4.1.1 Mechanical pain

Mechanical pain was assessed using a pressure algometer (Commander Algometer, JTECH Medical, Midvale, Utah), which was applied perpendicularly to the mid-phalanx of the right middle finger. using a pressure algometer, with measurements taken for both pain threshold (when pain was first felt) and pain tolerance (when pain became intolerable). Pressure increased at a rate of ∼9 N/cm²/s, and the pain threshold (“PressureThr”) and tolerance (“PressureTol”) were determined as the median of five repetitions each.

#### 4.1.2 Thermal cold pain

The thermal cold pain threshold (“TSACold”) was measured using a 3 × 3 cm² thermode (Thermal Sensory Analyzer, Medoc Advanced Medical Systems Ltd., Ramat Yishai, Israel) on the medial volar forearm. The thermode cooled from 32 °C to 0 °C at a rate of 1 °C/s, and the threshold was determined by taking the median of five trials.

#### 4.1.3 Infrared laser pain

Infrared laser stimuli were delivered to the dorsum of the left hand using a thulium solid-state laser (Themis®, StarMedTec GmbH, Starnberg, Germany) in 1 ms pulses with a 5 mm² area and 10 stimuli with an interstimulus interval (ISI) of 15–25 s. The pain threshold (“LaserThr”) was determined using a staircase method with pulse energy adjusted based on subject responses. The threshold was the mean of the highest nonpainful and first painful stimulus strengths. Pain intensity was rated for 23 stimuli at 650 mJ (“LaserVAS”) on a 100-mm visual analog scale (VAS) ranging from zero, “no pain” to 100, “maximum pain”. These procedures followed established lab standards.

#### 4.1.4 Electrical pain

The electrical pain threshold (“ElectricThr”) and tolerance (“ElectricTol”) were determined on the left mid-phalanx using a Neurometer® CPT device (Neurotron Inc., Baltimore, MD). The electrical current was increased from 0 to 20 mA at a rate of 0.2 mA/s. Subjects released a button at the first sign of pain or when pain became intolerable. Medians were recorded from five trials.

#### 4.1.5 Chemical (CO_2_) pain

Chemical pain was induced by 200-ms pulses of gaseous CO₂ at varying concentrations (30%−60% v/v), which were delivered to the nasal mucosa via a Teflon tube embedded in a constant air stream (8 L/min, 36.5 °C, 80% humidity), using an olfactometer (OM/2, Burghart Instruments, Wedel, Germany) [10]. These stimuli convert CO₂ into bicarbonate and protons via the enzyme carboanhydrase, which is expressed at the nasal mucosa [11]. This process evokes a short, stinging pain sensation [12]. During the experiments, subjects indicated whether each of the sixteen stimuli, applied in random order within the aforementioned CO₂ concentration range, was painful. The CO₂ pain threshold (“CO2Thr”) was obtained by averaging the highest nonpainful concentration and the lowest painful concentration. This method yielded results that agreed with the binary logistic regression analysis of CO₂ concentration versus yes/no responses for pain. Pain intensity was rated for 23 stimuli at 65% CO₂ using a 100-mm visual analog scale (“CO2VAS”).

### 4.2 Standardized quantitative sensory testing (QST) battery

A standardized clinical QST test battery was used to assess pain sensitivity. This battery was established by the German Research Network on Neuropathic Pain (DFNS) [5,6], encompassing 11 tests, yielding 13 parameters, and were performed in a fixed order on randomly assigned sites (dorsal hand or foot) [13]. Thermal thresholds (detection and pain) were measured using thermodes with controlled ramp rates, testing for cold and warm sensitivity, thermal sensory limen (TSL), and paradoxical heat sensations. Mechanical thresholds were assessed using von Frey filaments and calibrated pinprick stimulators for mechanical detection threshold (MDT), mechanical pain threshold (MPT), and mechanical pain sensitivity (MPS), including tests for dynamic mechanical allodynia (DMA). Temporal pain summation (wind-up ratio, WUR) was evaluated using repeated pinprick stimuli. Vibration detection threshold (VDT) was determined with a tuning fork. PPT was measured using a pressure algometer at defined ramp rates until the subject reported pain. For further details, see Table 2.

Room temperature was maintained at 20–25 °C. A trained investigator conducted all measurements in accordance with published protocols [6,14]. The tested body area was randomly assigned (dorsal hand or foot), and the VDT and PPT were measured at specific anatomical landmarks. The initial processing of the raw data followed the published guidelines and included log transformation and z-transformation of the 11 QST parameters using the reference values from a healthy cohort [15]. The sign was adjusted to indicate high or low sensitivity. Paradoxical heat sensations and DMA were excluded from the z-transformation due to the unavailability of reference data [16].

#### 4.2.1 Thermal detection and pain thresholds

Cold detection threshold (CDT), warm detection threshold (WDT), thermal sensory limen (TSL, including paradoxical heat sensations, PHS), cold pain threshold (CPT), and heat pain threshold (HPT) were measured using a computer-controlled thermode (9 or 12.5 cm²; baseline 32 °C; ramp 1 °C/s; cutoffs 0–5 °C for cold, 50 °C for heat). Subjects pressed a button at the first perception of a temperature change or pain. Thresholds were averaged over three trials. TSL involved alternating warm and cold stimuli, and subjects indicated the direction of the stimuli as well as any PHS (warmth or burning on cooling).

#### 4.2.2 Mechanical detection and pain thresholds

The mechanical detection threshold (MDT) was assessed using von Frey filaments (0.25–512 mN), and the mechanical pain threshold (MPT) was assessed using calibrated weighted pinprick stimulators (8–512 mN). Thresholds were determined using the method of limits and calculated as the geometric mean of just sub- and suprathreshold intensities.

#### 4.2.3 Mechanical pain sensitivity (MPS) and dynamic mechanical allodynia (DMA)

MPS was tested by applying pinprick stimuli of varying intensities five times, and subjects rated the pain on a scale from 0 to 100. Dynamic mechanical allodynia (DMA) was assessed by gently stroking the skin with a brush, Q-tip, or cotton swab, and subjects rated any pain elicited by these normally nonpainful stimuli.

#### 4.2.4 Wind-up ratio (WUR)

Temporal summation of pain was evaluated by comparing pain ratings for a single stimulus versus a series of ten repetitive stimuli (1 Hz) using a 256-mN pinprick stimulator. The WUR was calculated by dividing the mean pain rating for the series by the mean rating for single stimuli.

#### 4.2.5 Vibration detection threshold (VDT)

The VDT was measured by applying a 64-Hz tuning fork to a bony prominence. Subjects indicated when they could no longer perceive the vibration, and the mean of three trials was recorded.

#### 4.2.6 Pressure pain threshold (PPT)

The PPT was determined by applying a pressure algometer at a constant rate of 0.5 kg/s until the subject reported the first sensation of pain. The mean of three measurements was used.

## Limitations

A limitation of the data is that the QST parameters were acquired only in healthy subjects from untreated skin. This particularly impacts three QST parameters that have nearly identical values in all subjects. The respective parameters are (i) paradoxical heat sensations (PHS; perception of warmth during alternating cooling and warming), (ii) vibration detection threshold (VDT; minimum detectable vibration intensity), and (iii) dynamic mechanical allodynia (DMA; pain from normally nonpainful stroking touch). These measures are relevant for diagnosing neuropathic conditions. That is, PHS reflects altered thermal perception, VDT assesses sensory nerve function, and DMA is a marker of central sensitization that is clinically significant in disorders.

As investigator identity was not recorded, precluding formal assessment of inter-investigator variability.

The dataset contains 96 missing values (3.3% of all QST values). For many analyses, especially those using machine learning, imputation is necessary to address these gaps. If imputation is not performed or desired as in [1], removing incomplete cases reduces the sample size to n = 72 because the missing values are distributed across the dataset.

## Ethics Statement

This data set is from a previously published study that had been performed in accordance with the Declaration of Helsinki and was approved by the Ethics Committee of the Medical Faculty of the Goethe University Frankfurt am Main, Germany (reference number 27/11 and 71/12). The responsible ethics review board has explicitly approved the upload of the anonymized data to a public repository.

## CRediT Author Statement

**Jörn Lötsch:** Conceptualization, Methodology, Software, Validation, Resources, Data Curation, Writing - Original Draft, Writing - Review & Editing, Supervision, Project administration, Funding acquisition, Revision of the manuscript; **Violeta Dimova:** Project administration, Methodology, Investigation, Writing - Review & Editing; **Isabel Lieb:** Investigation, Writing - Review & Editing; **Dario Kringel:** Data Curation, Writing - Original Draft, Writing - Review & Editing.

## Data Availability

Mendeley DataQuantitative sensory testing and classical pain model dataset in 127 healthy volunteers (Original data).

## References

[1] Lötsch J., Dimova V., Ultsch A., Lieb I., Zimmermann M., Geisslinger G., Oertel B.G (2016). A small yet comprehensive subset of human experimental pain models emerging from correlation analysis with a clinical quantitative sensory testing protocol in healthy subjects. Eur. J. Pain.

[2] Lötsch J., Dimova V., Hermens H., Zimmermann M., Geisslinger G., Oertel B.G., Ultsch A. (2015). Pattern of neuropathic pain induced by topical capsaicin application in healthy subjects. Pain.

[3] Lötsch J., Oertel B.G., Ultsch A. (2014). Human models of pain for the prediction of clinical analgesia. Pain.

[4] Oertel B.G., Lötsch J. (2013). Clinical pharmacology of analgesics assessed with human experimental pain models: bridging basic and clinical research. Br. J. Pharmacol..

[5] Rolke R., Baron R., Maier C., Tolle T.R., Treede R.D., Beyer A., Binder A., Birbaumer N., Birklein F., Botefur I.C., Braune S., Flor H., Huge V., Klug R., Landwehrmeyer G.B., Magerl W., Maihofner C., Rolko C., Schaub C., Scherens A., Sprenger T., Valet M., Wasserka B. (2006). Quantitative sensory testing in the German Research Network on Neuropathic Pain (DFNS): standardized protocol and reference values. Pain.

[6] Rolke R., Magerl W., Campbell K.A., Schalber C., Caspari S., Birklein F., Treede R.D (2006). Quantitative sensory testing: a comprehensive protocol for clinical trials. Eur. J. Pain.

[7] Lötsch J., Mayer B., Kringel D. (2023). Machine learning analysis predicts a person's sex based on mechanical but not thermal pain thresholds. Sci. Rep..

[8] Fechner G.T (1860).

[9] Hummel T., Kobal G. (1999). Chemosensory event-related potentials to trigeminal stimuli change in relation to the interval between repetitive stimulation of the nasal mucosa. Eur Arch. Otorhinolaryngol..

[10] Kobal G. (1985). Pain-related electrical potentials of the human nasal mucosa elicited by chemical stimulation. Pain.

[11] Tarun A.S., Bryant B., Zhai W., Solomon C., Shusterman D. (2003). Gene expression for carbonic anhydrase isoenzymes in human nasal mucosa. Chem. Senses.

[12] Hummel T., Mohammadian P., Marchl R., Kobal G., Lötsch J. (2003). Pain in the trigeminal system: irritation of the nasal mucosa using short- and long-lasting stimuli. Int. J. Psychophysiol..

[13] Pfau D.B., Krumova E.K., Treede R.-D., Baron R., Toelle T., Birklein F., Eich W., Geber C., Gerhardt A., Weiss T., Magerl W., Maier C. (2014). Quantitative sensory testing in the German Research Network on Neuropathic Pain (DFNS): reference data for the trunk and application in patients with chronic postherpetic neuralgia. Pain.

[14] Pfau D., Klein T., Blunk J.A., Geber C., Krumova E., Limbeck C., Magerl W., Maier C., Westermann A., Schuh-Hofer S., Tiede W., Treede R.D, Rolke R., Andrews A., Magerl W., Treede R.D. (2010). Lehrstuhl Für Neurophysiologie.

[15] Magerl W., Krumova E.K., Baron R., Tolle T., Treede R.D., Maier C. (2010). Reference data for quantitative sensory testing (QST): refined stratification for age and a novel method for statistical comparison of group data. Pain.

[16] Maier C., Baron R., Tölle T.R., Binder A., Birbaumer N., Birklein F., Gierthmühlen J., Flor H., Geber C., Huge V., Krumova E.K., Landwehrmeyer G.B., Magerl W., Maihöfner C., Richter H., Rolke R., Scherens A., Schwarz A., Sommer C., Tronnier V., Uçeyler N., Valet M., Wasner G., Treede R.-D (2010). Quantitative sensory testing in the German Research Network on Neuropathic Pain (DFNS): somatosensory abnormalities in 1236 patients with different neuropathic pain syndromes. Pain.

